# Genome reorganization in different cancer types: detection of cancer specific breakpoint regions

**DOI:** 10.1186/s13039-019-0435-3

**Published:** 2019-06-20

**Authors:** Christoph Standfuß, Jonas Parczyk, Jerome Ruhnau, Andreas Klein

**Affiliations:** 1 Institute of Biochemistry, Charité – Universitätsmedizin Berlin, Corporate Member of Freie Universität Berlin, Humboldt-Universität zu Berlin, and Berlin Institute of Health, Charitéplatz 1, 10117 Berlin, Germany; 2 Institute of Biochemistry, Charité – Universitätsmedizin Berlin, corporate member of Freie Universität Berlin, Humboldt-Universität zu Berlin, and Berlin Institute of Health, MTU Aero Engines, 80995 Munic, Germany

**Keywords:** Pancreatic ductal adenocarcinoma, Breast cancer, Melanoma, Copy number variation, Cancer genomics, Breakpoint and genome reorganization

## Abstract

**Background:**

Tumorigenesis is a multi-step process which is accompanied by substantial changes in genome organization. The development of these changes is not only a random process, but rather comprise specific DNA regions that are prone to the reorganization process.

**Results:**

We have analyzed previously published SNP arrays from three different cancer types (pancreatic adenocarcinoma, breast cancer and metastatic melanoma) and from non-malignant control samples. We calculated segmental copy number variations as well as breakpoint regions. Some of these regions were not randomly involved in genome reorganization since we detected fifteen of them in at least 20% of all tumor samples and one region on chromosome 9 where 43% of tumors have a breakpoint. Further, the top-15 breakpoint regions show an association to known fragile sites. The relevance of these common breakpoint regions was further confirmed by analyzing SNP arrays from 917 cancer cell lines.

**Conclusion:**

Our analyses suggest that genome reorganization is common in tumorigenesis and that some breakpoint regions can be found across all cancer types, while others exclusively occur in specific entities.

## Background

Tumorigenesis is a stepwise process, which involves multiple genetic, epigenetic and genomic events to transform a normal cell into a tumor cell [1–6].Genomic changes like copy number variations (CNVs) or segmental copy number variations (segCNVs) increase throughout tumorigenesis [7–9] and are caused by various mechanisms, like fork stalling during replication or nonallelic homologous recombination [10–12].

These changes can affect the chromatin structure and therefore the spatial localization of specific genes, the DNA sequence like single nucleotide mutations, amplifications, deletions or translocations as well as changes of karyotypes like aneuploidies [1, 13–16].

It is also speculated that DNA regions exist which are prone for reorganization. Pevzner and Tesler stated in their seminal work “that mammalian genomes are mosaics of fragile regions with high propensity for rearrangements and solid regions with low propensity for rearrangements [17].”

Their thesis stands in contrast to the established theory of the random breakage model. The latter is based on the following two assumptions: Chromosomal segments are conserved among different species and chromosomal rearrangements are randomly distributed within the genome [18]. Indeed, it is well established that chromosomal segments exist in different species where orthologous genes are located in the same arrangement. On the other hand, it is now also established that specific DNA regions throughout the genome are prone to breakage and reorganization [17, 19–21]. Ruiz-Herrera et al stated that “certain chromosomal regions in the human genome have been repeatedly used in the evolutionary process. As a consequence, the genome is a composite of fragile regions prone to reorganization…” Well known regions exhibiting chromosomal instability are fragile sites, which were firstly described by Magenis et al 1970 [22, 23]. “Fragile sites are specific loci that form gaps, constrictions, and breaks on chromosomes exposed to partial replication stress and are rearranged in tumors [24].” Fragile sites can be divided in rare and common fragile sites (CFSs). Rare fragile sites are only expressed in a few individuals. They are associated with the expansion of micro- or minisatellite repeats and inheritable diseases like fragile X syndrome. CFSs are regular parts of chromosomes and therefore found in all humans. CFSs are hotspots for metaphase chromosomal gaps and breaks and chromosomal rearrangements. CFS instability is an early step in tumorigenesis and could be responsible for genome reorganization in cancer [23, 25–29].

In 2012, Standfuß et al observed the stepwise increase in genome reorganization in a simian virus 40 (SVT/t) transformed mouse breast cancer model. The number of genomic changes increased from non-malignant, to hyperplastic and to tumor samples of mammary glands. Moreover, distinct breakpoint regions, where genome reorganization events take place, could be detected. They argued that unique and common breakpoint regions exist in breast cancer. However, due to the small sample size, the final proof was missing [9].

In this study, we analyzed DNA SNP arrays from 20 healthy controls and 111 cancer samples as well as 917 cancer cell lines. We found unique and common breakpoint regions in different cancer entities and more strikingly, we found a breakpoint region which was common in more than one third of all tumors and cancer cell lines tested.

Thus, we addressed the questions, whether genome reorganization is a random process, and whether specific DNA regions are prone to this reorganization procedure.

## Material and methods

### SNP array data

We reanalyzed 131 single-nucleotide polymorphism (SNP) microarrays, produced using the Genome-Wide Human SNP Array 6.0 platform (https://tools.thermofisher.com/content/sfs/brochures/genomewide_snp6_datasheet.pdf). The 111 tumor samples compromise 25 pancreatic adenocarcinomas (PDAC) from Donahue et al [30] [GSE32688], 22 PDAC derived cell lines from Barretina et al [31] [GSE36139], 16 metastatic melanomas from Marzese et al [32] [GSE44019] and 48 breast cancer samples from [GSE26232]. The 20 non-malignant control experiments (NMCE) compromise 15 samples derived from B cells isolated from peripheral blood of healthy donors from Xie et al [33] [GSE49045] and 5 samples derived from peripheral blood cells of breast cancer patients [GSE48377]. The 15 blood samples from healthy donors were further termed as “reference” and the five peripheral blood cells from breast cancer patients were termed as “control”.

Further, we analyzed 917 cancer cell line samples from the Cancer Cell Line Encyclopedia (CCLE) [31] [GSE36139]. All samples are publicly available.

### Copy-number variation

Raw SNP microarray data was processed using the Affymetrix Power Tools 1.15.0 (now Oncomine™ Power Tools, Thermo Fisher Scientific) and the BRLMM-P algorithm to extract the normalized SNP signal intensities. To compare the total signal intensity distributions of all samples, intensities of both alleles for each SNP were added up. CNVs for each SNP was computed as log2-ratios of each tumor sample and the reference dataset comprising 15 blood samples from healthy donors. The reference for each SNP was calculated as the average signal intensity of the 15 reference samples.

SegCNVs for each sample were computed with the DNAcopy package (1.36.0) of Bioconductor (2.13) [34] with the following parameters: alpha = 0.001, undo.splits = “sdundo”, undo.SD = 0.5, min.width = 4. The DNAcopy package implements the circular binary segmentation algorithm introduced by Olshen et al [35]. The number of segCNVs were counted for each experiment and set in relation to the number of base pairs for each chromosome. We excluded Chromosome Y (860 SNPs) and MT (411 SNPs) from our analyses. The heat map was generated using ggplot2 package of R. Hg19, provided by the University of California, Santa Cruz (UCSC), was used for human genome assembly.

### Common breakpoints

The genome was divided into 30,951 bins of 100 kb size or less, if the bin represents a chromosomal end region. The occurrence of each breakpoint was counted in all 1048 analyzed samples to find regions of predisposed alterations. To enhance stringency, a breakpoint between two segCNVs was defined as follows: 1) the log2-ratio difference between both segments has to be greater than 0.5. 2) at least one segment has to include a minimum of 10 and the other of 4 SNPs.

### Odds ratio

To decide whether a breakpoint event (BP) is more frequent in cancer samples than in the NMCE, we calculated the odds ratios.

oddsNMCE = (number of NMCE with BP)/(total number of NMCE - number of NMCE with BP).

oddsTumor = (number of tumors with BP)/(total number of tumors - number of tumors with BP).

oddsRatio = (oddsTumors)/(oddsNMCE)

Since some of the breakpoints were not found in the NMCE but had a high count in the tumor group odds ratio, calculations were not trivial. In accordance with the Cochrane Handbook for Systematic Reviews of Interventions we added 0.5 in those cases:

oddsNMCE = (number of NMCE with BP + 0.5)/ (total number of NMCE + 0.5 - number of NMCE with BP + 0.5).

oddsTumor = (number of tumors with BP + 0.5)/(total number of tumors + 0.5 - number of tumors with B + 0.5).

oddsRatio = (oddsTumors)/(oddsNMCE)

### Fragile sites

We used the chromosomal location of the 230 fragile sites published by Mrasek et al [36] and analyzed their occurrence within our breakpoint regions. Therefore, the cytogenetic location was translated into the chromosomal location with the help of the “Ensemble Genome Browser version GRCh37.p13.”

## Results

### SNP CNVs in different tumor entities

To study the changes in genome reorganization during tumorigenesis, we analyzed previously published SNP arrays from 111 cancer samples: 25 pancreatic ductal adenocarcinoma, 22 PDAC derived cell lines, 16 metastatic melanoma and 48 breast cancer samples. As NMCE, we used DNA from peripheral blood samples from healthy donors and from breast cancer patients.

**Fig. 1:**
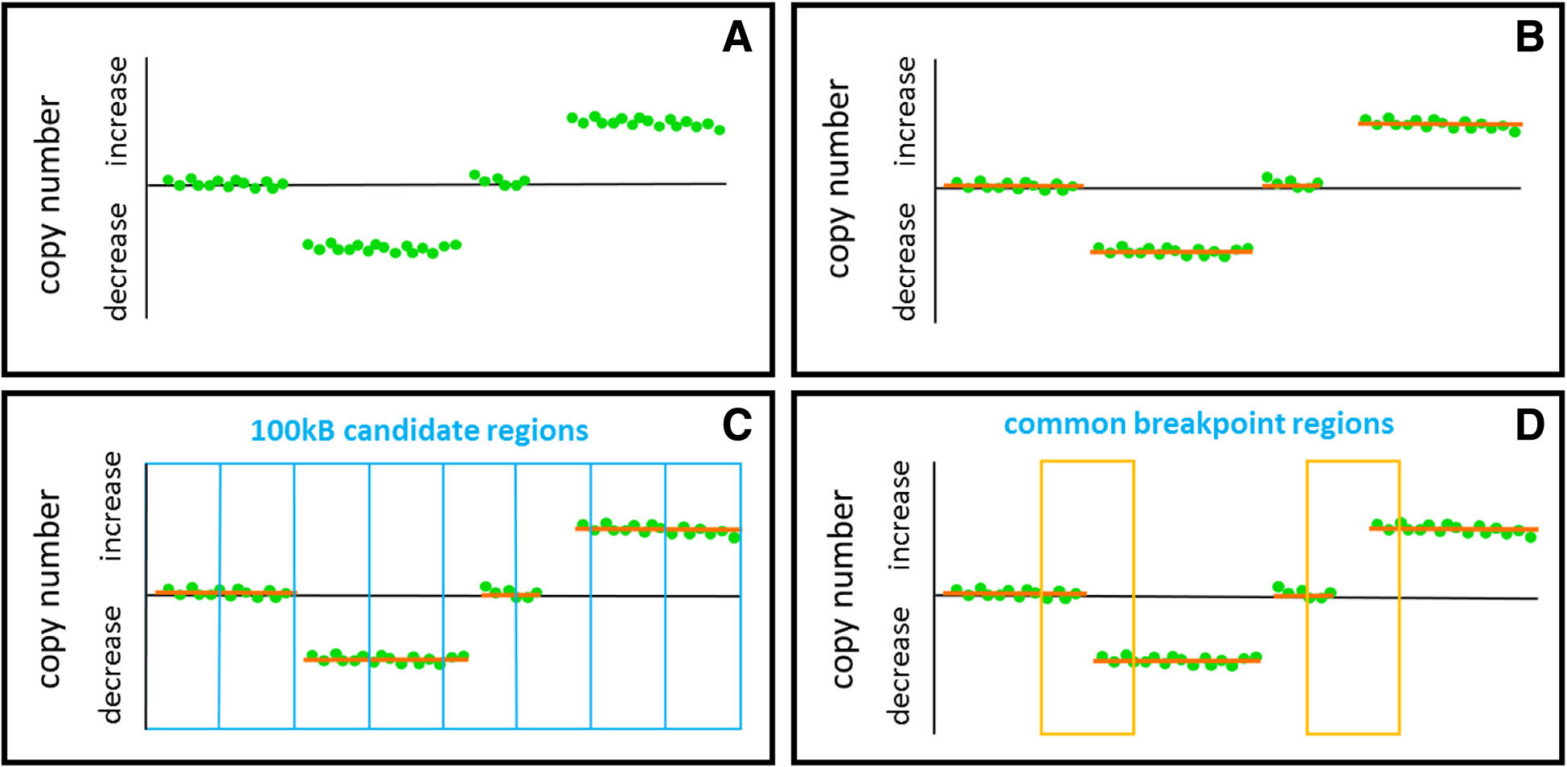
Illustration of the experimental approach for detecting common breakpoint regions. We computed SNP-CNV - green dots - for each chromosome (**a**) and computed segments of similar copy-number - red segments (**b**). To assess regions with frequent chromosomal aberrations, we divided each chromosome into candidate regions of 100 kb size (**c**). Within each 100 kb bin, we counted each beginning of a new segCNV with difference in log2-ratio of 0.5 as a breakpoint. Breakpoint regions with counts in multiple samples (**d**) were considered as common breakpoint regions and further analyzed

We added up the signal intensities for SNP alleles and further determined continuous SNP CNV regions for all chromosomes using the circular binary segmentation algorithm introduced by Olshen and colleagues [35]. In order to define DNA regions with a high probability of genomic reorganization and which were common in multiple cancer samples, we divided the genome into 30,951 bins of 100 kb size and defined a breakpoint region as follows: at least two DNA segments have to differ in their average copy number values of more than a log2-ratio of 0.5 and one segment has to consist of 10 SNPs instead of the minimum of four SNPs. Thus, breakpoint regions were defined as DNA sites where segmental copy number level shifts occur. If a breakpoint is present in multiple tumor samples, we call it common breakpoint region. This approach is illustrated in Fig. 1.

**Fig. 2:**
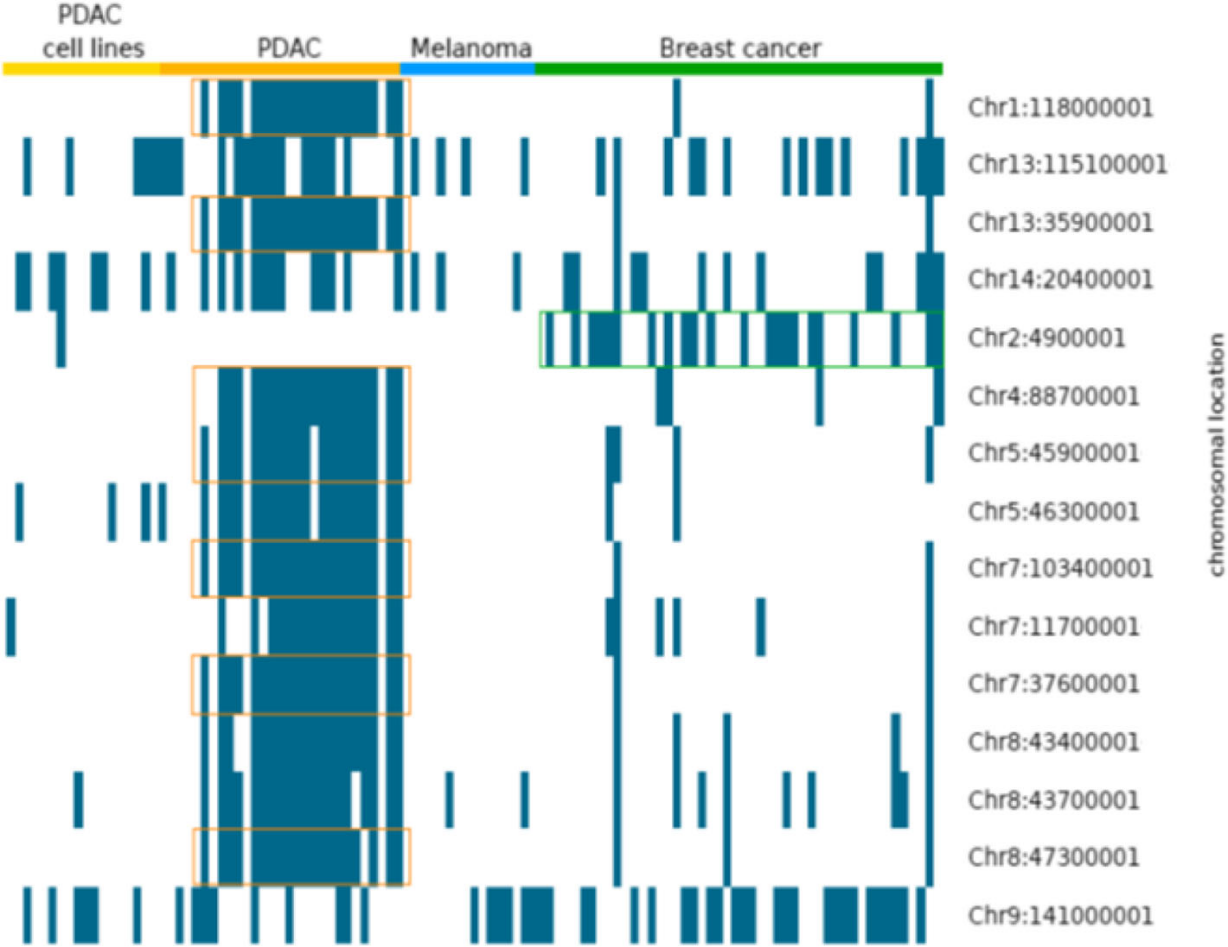
To illustrate the presence (blue line) of common breakpoints within different tumor samples and tumor entities, we created a heat map. The chromosomal location is listed on the y-axis. Here, we present a heat map for all common breakpoints that appear in at least 20% of tumor samples. Breakpoints that were mainly common to PDAC tumor samples are marked by orange rectangles and green rectangles mark breakpoints common to breast cancer samples

In total, we found 19,687 regions (63.61%) where at least one experiment had a breakpoint. However, since most of the breakpoint regions were present in only one or two tumor samples, we focused on genomic regions in which at least 23 out of the 111 tumors (20%) had a breakpoint (Fig. 2, Table 1). The heat map shows the fifteen 100 kb sized breakpoint regions, which appear in at least 20% of all tumor samples. We highlighted breakpoints more frequent in PDAC tumor samples with orange boxes, and regions more frequent in breast cancer samples with green boxes. This result indicates that some breakpoints are more frequent in only one tumor entity (like chromosomes 1, 2 and 13) whereas other regions are present in all tumor entities (like chromosomes 9 and 13). The breakpoints on Chromosomes 9 and 13 had 43 and 36% of all tumors in common. Since some breakpoints were also present in the NMCE, we verified the relevance of a breakpoint region by determining the odds ratio for being tumor specific.

**Table 1:** Chromosomal location, occurrence of breakpoint events (BP), odds ratio, located genes and association to fragile sites of the top-15 breakpoint regions. Genes that are associated with cancer in literature are marked with an asterisk

Chr	Start	End	Cytoband	BP in NMCE (20)	BP in Tumors (111)	Odds Ratio	BP in CCLE (917)	Genes	Fragile Sites
1	118000001	118100001	1p12	0	23	10,68	4	*MAN1A2*	–
2	4900001	5000001	2p25.2	0	23	10,68	26	*–*	FRA2M
4	88700001	88800001	4q22.1	0	24	11,26	2	IBSP*, MPEP*	FRA4F
5	45900001	46000001	5p12	0	24	11,26	1	–	–
5	46300001	46400001	5p11	0	26	12,47	81	–	FRA5I
7	11700001	11800001	7p21.3	0	24	11,26	5	THSD7A*	FRA7L
7	37600001	37700001	7p14.1	0	23	10,68	0	*NECAP1P1*	–
7	103400001	103500001	7q22.1	1	23	4,97	1	RELN*	FRA7F
8	43400001	43500001	8p11.1	0	25	11,86	2	–	FRA8I
8	43700001	43800001	8p11.1	0	32	16,46	48	–	FRA8I
8	47300001	47400001	8q11.1	0	23	10,68	1	–	FRA8I
9	141000001	141100001	9q34.3	1	48	14,48	321	CACNA1B*	FRA9N
13	35900001	36000001	13q13.3	0	23	10,68	1	NBEA*	–
13	115100001	115106996	13q34	3	40	3,19	210	–	FRA13I
14	20400001	20500001	14q11.2	3	36	2,72	61	*OR4K1*, *OR4K5*, *OR4K14*, *OR4K15*	FRA14D

Table 1 shows the odds ratio for the breakpoints illustrated in Fig. 2. In all of the top-15 breakpoint regions, we observed that on average, an odds ratio > 10 indicates a high prevalence for these breakpoints to occur in tumor samples. The two highest odds ratio values were calculated for the breakpoint of chromosome 9 present in 48 different tumor samples and one NMCE (odds ratio = 14.5) and the breakpoint on chromosome 8 (43,700,001) present only in 32 different tumor samples (odds ratio = 16.5). Twelve genes were located in eight of the top-15 breakpoint regions, and six of these genes are associated with cancer (*CACNA1B, IBSP, MEPE, NBEA, RELN and THSD7A*) (Table 1).

### Cancer cell line encyclopedia (CCLE)

To further validate, the top-15 breakpoint regions, we included 917 cancer cell line samples in our analyses. We summarized in Table 2 the seven 100 kb sized breakpoint regions which appear in at least 20% of all CCLE samples The breakpoint regions on Chromosomes 9 (141,000,001) and 13 (115,100,001) which were present in all tumor entities, also had the most breakpoints in the analyzed cancer cell lines. On Chromosome 9, 321 cancer cell lines (34%) and on Chromosome 13, 210 (22%) cancer cell lines had a breakpoint within the aforementioned regions. Five genes were located in four of the seven breakpoint regions and three of these genes (*CACNA1B, C8orf33 and KIAA0513*) are associated with cancer (Table 2). Interestingly, only very few cancer cell lines (< 0.5%) had breakpoints in the seven breakpoint regions that were associated with PDAC: e.g. the region on chromosome 7 (37,600,001) had no breakpoint in cancer cell lines and the regions on chromosomes 5 (45,900,001), 7 (103,400,001), 8 (47,300,001) and 13 (35,900,001) had only one breakpoint in cancer cell lines (Table 1). The breast cancer associated breakpoint region on chromosome 2 is also only shared by 2.8% of cancer cell lines.

**Table 2:** Chromosomal location, occurrence of breakpoint events (BP), odds ratio, located genes and association to fragile sites of the top-ranked CCLE breakpoint regions. Genes that are associated with cancer in literature are marked with an asterisk. Interestingly, the breakpoint region in chromosome 2 is close to the cancer associated *SDC1* gene by about 558 bases

Chr	Start	End	Cytoband	BP in NMCE (20)	BP in Tumors (111)	Odds Ratio	BP in CCLE (917)	Gene	Fragile Sites
2	20300001	20400001	2p24.1	0	9	3,73	248	–	–
4	190900001	191000001	4q35.2	0	14	5,98	190	*FRG2*	FRA4L, FRA4M
7	159100001	159119220	7q36.3	0	22	10,11	230	–	FRA7I
8	146200001	146300001	8q24.3	0	21	9,56	238	C8orf33*	FRA8D
9	141000001	141100001	9q34.3	1	48	14,48	321	CACNA1B*	FRA9N
13	115100001	115106996	13q34	3	40	3,19	210	–	FRA13I
16	85000001	85100001	16q24.1	0	8	3,30	244	KIAA0513*, *ZDHHC7*	FRA16J

The presented results indicate that we created a set of common breakpoint regions with the help of PDAC, melanoma metastasis and breast cancer samples that were more highly associated with single cancer entities, whereas other breakpoint regions can be found in a variety of tumors.

### Fragile site

Since fragile sites are well known regions exhibiting chromosome instability, we compared the chromosomal locations of the common breakpoint regions we found with data from chromosomal fragile sites [36]. Eleven thousand three hundred sixty out of the 19,687 breakpoint regions contained a fragile site (58%).

Since an odds ratio less than one indicates a higher likelihood of a breakpoint region to occur in NMCE, and an odds ratio above one indicates higher odds for occurring in tumor samples, we determined the percentage of a fragile site to occur in relation to the odds ratio. Out of the 19,687 breakpoint regions, 13,063 had an odds ratio of less than one and 6624 above one. A region with an odds ratio < 1 occurred in 57% (7471 out of 13,063) associated with fragile sites and a region with an odds ratio > 1 occurred in 59% (3889 out of 6624) associated with fragile sites. Thus, we could not determine a crucial difference in the association to fragile sites in the more tumor linked breakpoint regions.

However, 11 of the top-15 breakpoint regions (73%) were associated with fragile sites and 6 of the 7 CCLE related breakpoint region (86%), indicating a strong association of the top-ranked breakpoint regions to known fragile sites.

### Targeted investigation

Further, we evaluated important regions known for genome reorganization from literature (e.g. loss-of heterozygosity or homozygous deletion) and looked for the relevance of those regions in our dataset concerning the occurrence of breakpoints. Fragile site FRA16D (16q23.2) is within a region of frequent loss-of-heterozygosity in breast and prostate cancers. Interestingly, we found 64 breakpoints in 13 tumor samples (11.7%) for this fragile site, whereof 61 were found in nine breast cancer samples (18.75% of all breast cancer samples). Another frequently altered chromosomal region is located on chromosome 9 (21,900,001) where the *tumor suppressor p16* (official symbol *CDKN2A*) is present. In the corresponding bins, 104 cancer cell lines had a breakpoint (11.34%) and eight tumor samples (7.2%). Interestingly, this region is part of the fragile site FRA9A. In this CFS 56 tumor samples (50.5%) had at least one breakpoint.

The most commonly known unstable CFS region is FRA3B [37]. In this CFS, spanning over 43 bins, 148 breakpoints were detected in 26 cancer samples (23.4%). It is also noteworthy that 23 out of the 26 cancer samples had a breakpoint in the region of the gene *FHIT* lying inside of FRA3B. In line with this, 243 cancer cell lines have breakpoints in FRA3B and 223 of those have breakpoints in the 16 bins containing *FHIT*.

## Discussion

In this study, we examined the theory that genome reorganization during tumorigenesis is not a random process but rather a directed process, involving defined DNA regions. Therefore, we have reanalyzed 1.048 DNA SNP arrays from different cancer entities and non-malignant samples. We found an increase of DNA breakpoint regions in tumor samples compared to NMCE. Interestingly, several breakpoint regions were common in several tumor specimen (up to 43%) where as other regions seemed to be more restricted to a specific tumor entity. Surprisingly, breakpoint regions between PDCA and PDCA derived cell lines differ considerably. On the one hand, Kalinina and colleagues established a pancreatic cancer cell line from a primary tumor. Kalinina and colleagues also observed a similar CNV pattern between tumor and cell line after passaging the cell line 15–20 times, as well as a considerable number of similar large chromosomal alterations [38]. On the other hand, Burdall and colleagues stated that “Cell lines are prone to genotypic and phenotypic drift during their continual culture. This is particularly common in the more frequently used cell lines, especially those that have been deposited in cell banks for many years [39].” This might be applicable for the used cell lines in our approach, e.g. Capan 1 and 2 were established 1974 and 1975, respectively [40, 41].

It is well known that cancers develop from stem lines in a stepwise process and are characterized by chromosomal aberrations and chromosomal instability [42, 43]. The Mitelman Database of Chromosome Aberrations and Gene Fusions in Cancer currently lists 69,134 human cancers with individual clonal karyotypes [44]. In 2012, Standfuß et al found a stepwise increase in genome reorganization in a mouse breast cancer model. The number of genomic changes increased from non-malignant, to hyperplastic and to tumor samples of mammary glands [9]. Further, an analysis of 2.737 tumor samples from 8 different tumor entities (including breast cancers) showed that tumor entity-specific breakpoints could be found for all examined tumor entities. The breakpoint regions were equally distributed over all entities [45]. Further, colocalization assessment identified 20,077 CNV-affecting genes and 169 of these being known tumor-related genes. In another study, Beroukhim et al looked for somatic CNVs in 3.131 cancer specimen and found 158 regions of focal somatic CNVs of which only 36 can be explained by the presence of known cancer target genes located within this region like *FHIT* and *p16* [8]. Meaburn and Misteli also identified several genes specifically repositioned during tumorigenesis. The alterations of the spatial positioning were unrelated to gene activity [15]. In our study, genes were located in eight of the top-15 and four of the top-7 CCLE breakpoint regions. Eight of these genes are linked to cancer, but none are well characterized oncogenes or tumor suppressor genes. Interestingly, only *C8orf33* and *NBEA* seemed to have tumor suppressor functions [46, 47]. The other six genes are associated with tumor progression. *IBSP*, *MEPE*, *RELN* and *THSD7A* are associated with migration, invasion, infiltration and angiogenesis [48–51]; *CACNA1B* and *KIAA0513* are associated with cell proliferation and apoptosis. *CACNA1B* overexpression is associated with an unfavorable prognosis in non-small cellular lung cancer [52] and altered expression of *KIAA0513*, due to an aberrant methylation pattern, correlated with non-survivors in Neuroblastoma [53].

As early as 1984, several scientists postulated an association between human fragile sites and cancer breakpoints [25, 26, 54]. CFSs in cancer were considered as regions of chromosomal instability and their associated genes are frequently deleted or rearranged in cancer cells [55]. Since we found a strong correlation of our top breakpoint regions with fragile sites, we were also interested to look for breakpoints in specific CFSs described in literature. Finnis and colleagues found out that the CFS FRA16D (16q23.2) is located within regions of frequent loss-of-heterozygosity in breast and prostate cancers [56]. Here we found a breakpoint almost specific for breast cancer, since 61 from 64 breakpoints stem from breast cancer samples. 1986 Smeets and colleagues described FRA3B as the most unstable CFS region within chromosomal band 3p14.2 [37]. This chromosomal region is a hot-spot for deletions and other alterations in a variety of different cancers. *FHIT,* a large tumor suppressor gene spanning over approximately 35% of this fragile site, is also harbored in this region [57]. While 26 tumors and 243 cancer cell lines have a breakpoint in FR3B, the majority of these breakpoints, namely 23 and 223, lay in the *FHIT* gene. Thus, it is not surprising that estimates designate *FHIT* as the most frequently altered gene in cancer [58]. Inside the CFS, FRA9A the *p16* gene is located. Cox and Colleagues found in their “survey of homozygous deletions in human cancer genomes” that *p16* was the most frequent target of homozygous deletions (24.6%) [59]. Further, they argued that genetic rearrangement in this region might signify less negative selection compared to other regions because *p16* is located adjacent to one of the largest gene-poor regions of the human genome. When looking at the direct adjacent bins of *p16*, it stands out that the area of and around *p16* is the area of FRA9A where most of the breakpoints occur. This indicates that those breakpoints occurring in this CFS might play a role for tumor development, instead of being a random side effect of genomic instability.

However, genome rearrangements are not restricted to cancer cells. Rather, they are also present in adaptive processes, such as response to selective pressures from the environment and are associated with various diseases [60–62].

## Conclusion

In this study, we found that genome reorganization is more enhanced in tumor samples compared to the non-malignant controls and that some genome regions exist that are prone to rearrangements. We identified regions which may play an important role in the tumorigenesis of specific tumor entities and others that occur commonly during tumorigenesis.

For further investigations, genomic profiles could be linked to clinical data in order to produce additional prognostic markers for clinical outcome.

## Abbreviations

BP: breakpoint event
CCLE: Cancer Cell Line Encyclopedia
CFS: common fragile site
CNV: copy number variation
NMCE: non-malignant control experiment
PDAC: pancreatic adenocarcinoma
segCNV: segmental copy number variation
SNP: single-nucleotide polymorphism
